# Could local surgery improve survival in de novo stage IV breast cancer?

**DOI:** 10.1186/s12885-018-4767-x

**Published:** 2018-09-11

**Authors:** Zhenchong Xiong, Guangzheng Deng, Jin Wang, Xing Li, Xinhua Xie, Zeyu Shuang, Xi Wang

**Affiliations:** 1 Department of Breast Oncology, Sun Yat-sen University Cancer Center, 651 Dongfeng Road East, Guangzhou, 510060 China; 2 State Key Laboratory of Oncology in Southern China, Guangzhou, China; 3 Collaborative Innovation Center for Cancer Medicine, Guangzhou, China

**Keywords:** De novo stage IV, Local surgery, Bone metastasis, Soft tissue metastasis, Primary tumor size

## Abstract

**Background:**

Resection of the primary tumor is recommended for symptom relief in de novo stage IV breast cancer. We explored whether local surgery could provide a survival benefit in these patients and attempted to characterize the population that could benefit from surgery.

**Methods:**

Metastatic Breast cancer patients (*N* = 313) with intact primary tumor between January 2006 and April 2013 were separated into two groups according to whether or not they had undergone surgery. The difference in characteristics between the two groups was analyzed using chi-square test, Fisher’s exact test and Mann-Whitney test. Univariable and multivariable Cox regression and stratified survival analysis were used to assess the effect of surgery on survival.

**Results:**

Of the 313 patients, 188 (60.1%) underwent local surgery. Patients with local surgery had a 47% reduction in mortality risk vs. those with no surgery (median survival 78 months vs. 37 months; HR = 0.53; 95% CI, 0.36–0.78) after adjustment for clinical and tumor characteristics. Stratified survival analysis showed that patients with bone metastasis alone (and primary tumor ≤5 cm), soft tissue metastasis, or ≤ 3 metastasis sites benefit from surgery.

**Conclusion:**

Surgical resection of the primary tumor can improve survival in selected de novo stage IV breast cancer patients.

## Background

In 2012, about 1.7 million women were diagnosed with breast cancer worldwide, and it is estimated that 5–10% of them would have presented with de novo stage IV cancer [1–3]. As the advance of systemic treatments (endocrine therapy, cytotoxic therapy, anti-HER2 therapy, and so on) have greatly improved the control of metastatic diseases, local surgery may become feasible to improve survival combined with systemic therapy. Recent studies have suggested that resection of the primary tumor—a procedure that is usually reserved for palliative treatment—could strengthen local control and improve quality of life and progression-free survival in patients with metastatic breast cancer [4–8]. However, more convincing evidence to support the use of local surgery in stage IV breast cancer is required.

Several studies have shown that advanced local surgery may improve survival in women with metastatic breast cancer [2, 9–13]. Local resection of the primary tumor remains a positive prognostic factor in metastatic breast cancer even after adjustment for age, tumor burden, site of metastasis, race, type of surgery, margin status, and hormone receptor and HER2 receptor status [12–14]. A meta-analysis that included 28,693 patients with stage IV breast cancer found that patients who undergo surgical resection of the primary tumor have better survival than patients who do not undergo surgery [15]. Another group of researchers has suggested that patients with ER (estrogen receptor)/PR (progesterone receptor)-positive or HER2-amplified disease are most likely to benefit from local surgery [16]. One well-designed clinical trial from Turkey (NCT00557986) has shown that surgery significantly improves overall survival (OS) in patients with bone metastasis alone [17]. Although no survival benefit of local surgery was shown in patients with metastatic breast cancer, patients with local surgery were reported to have a better local control in a randomized control trial from India [4].

Due to clinical and biological heterogeneity in breast cancer, the exact cohort of patients who can be expected to benefit from local surgery is unknown. Outcomes in stage IV breast cancer vary with the site of metastasis, and this may provide a basis for identifying patients who may be likely to benefit from local surgery. Recent retrospective and clinical studies have consistently found that metastatic breast cancer patients with only bone metastasis benefit from local surgery [6, 13, 18]. However, it is uncertain whether the entire population of stage IV breast cancer patients with bone metastasis or only a subgroup of them should undergo local surgery. Whether patients with metastasis to other sites could benefit from surgery is also unknown. More studies examining the effects of surgery in different types of metastatic breast cancer patients are required to develop clinical practice guidelines.

The aim of this study was to determine if local surgery can improve OS in de novo metastatic breast cancer and to identify the characteristics of the patients who could be expected to benefit from surgery.

## Methods

### Patients

This retrospective study included 313 patients diagnosed with breast cancer at Sun Yet-sen University Cancer Center between 2006 and 2013. Patients were classified as stage IV breast cancer based on the seventh edition of the American Joint Committee on Cancer (AJCC) staging manual. Patients were eligible for inclusion if they had 1) pathological diagnosis of breast cancer; 2) metastatic disease at the time of diagnosis of breast cancer or developed metastasis within 3 months of diagnosis; and 3) life expectancy > 6 months. Patients were excluded if they 1) had incomplete follow-up data or 2) had history of previous cancer or synchronous malignant tumors. Ethical approval for the study was obtained from the institutional review board.

Information about pathological findings, age at diagnosis, type of surgery undergone, systemic therapy regimen, radiotherapy, and menstrual status (menopausal or premenopausal) was recorded. Time of death was recorded by clinic review or telephonic interview. Primary tumor status and metastasis status were regularly recorded by clinical and imageological examinations (mammography, ultrasound, and/or MRI for mammary examination; chest CT scan; positron emission tomography–computed tomography/PET-CT; and bone scan) at intervals of 2–4 months; patients with rapidly progressive disease or new symptoms were checked more frequently.

The enrolled patients were divided into two groups: a surgery group and a no surgery group. We defined four kinds of metastatic disease according to the sites involved: visceral metastasis (lungs, pleura, liver, and so on; yes/no), bone metastases (yes/no), soft tissues metastases (distant lymph nodes, skin, subcutaneous tissues; yes/no), and CNS metastases (brain, cranial nerves, and so on; yes/no). The number of metastatic sites was classified as ≤3 or > 3. HR status and ki-67 status are determined by Immunohistochemistry (IHC), and HER2 receptor status is determined by both IHC and fluorescence in situ hybridization (FISH). Tumor tissue containing over 1% of tumor cell which express estrogen receptor (ER)/progesterone receptor (PR) is defined as HR positive [19]. HER2 receptor amplification is defined as IHC (3+) or FISH (+) while others are defined as HER2 receptor non-amplification [20]. Ki-67 is assessed by calculating the proportion of tumor cells positively expressing Ki-67 [21]. Luminal A subtype is defined as HR positive, Ki-67 < 14% and HER2 receptor Non-amplification; Luminal B subtype is defined as HR positive, Ki-67 ≥ 14%/HER2 receptor amplification; HER2 subtype is defined as HER2 amplification and HR negative; Triple negative subtype is defined as HR negative and HER2 receptor Non-amplification [22]. Molecular subtype of breast cancer was categorized based on HR status, HER2 receptor status, and ki-67 status. OS was defined as the interval between the date of diagnosis and the date of death.

### Statistical analysis

Continuous variables were summarized as mean (± standard deviation) or median (and range), and were transformed into dichotomous variables at the median value. Comparisons were performed using the chi-square test or Fisher’s exact test for categorical variables and the Mann-Whitney test for continuous variables. The Kaplan-Meier method was used to analyze OS, and the log-rank test was used for comparisons between groups. Cox regression model was used to evaluate the association between clinical and tumor characteristics and OS. Individual covariates which significantly correlated with prognosis (*p* < 0.05) in the univariable model were entered into the multivariable model. Unadjusted and adjusted mortality risks (hazard ratios, with 95% CIs) were calculated. All *p* values were 2-sided, and *p* ≤ 0.05 were considered significant.

Patients were pre-stratified as factors: bone metastasis only (primary tumor ≤5 cm vs. primary tumor > 5 cm), visceral metastasis only (primary tumor ≤5 cm vs. primary tumor > 5 cm), soft tissue metastasis only and number of metastasis site (≤3 vs. > 3). Cox proportional hazards model was used to assess the mortality risk (hazard ratio and 95% CI) in each subgroup. The results were displayed in a forest plot.

## Results

### Patient characteristics

The study sample comprised 313 metastatic breast cancer patients with intact primary tumors. The median age of the patients was 47 years. Of the 313 patients, 188 (60.1%) patients received local surgery; 181/188 patients underwent modified radical mastectomy and 184/188 patients had achieved negative margin. The median follow-up duration was 25 months. Median survival was for 62 months. Table 1 shows the clinical and tumor characteristics.

**Table 1:** Characteristics of patients who were diagnosed with de novo stage IV breast cancer

Variable	No. Patients(*N* = 313)	Percentage
Age at diagnosis		
≤ 50	189	39.6
> 50	124	60.4
Menopause status		
Menses	193	61.7
Menopause	115	36.7
Unknown	5	1.6
Biopsy		
Mass resection	27	8.6
Core needle	229	73.2
Fine needle	1	0.3
Unknown	56	17.9
Tumor size		
≤ 5 cm	174	55.6
> 5 cm	120	38.3
Unknown	19	6.1
Lymph node involvement		
N_0_	28	8.9
N_1_	44	14.1
N_2_	71	22.7
N_3_	129	41.2
Unknown	41	13.1
Hormone receptor status		
+	212	67.7
-	94	30.0
Unknown	7	2.2
HER2 receptor status		
Amplified	132	42.2
No-amplified	161	51.4
Not known or equivocal(2+)	20	6.4
Molecular subtype		
Luminal A	55	17.6
Luminal B	141	45.0
HER2	56	17.9
Triple negative	33	10.5
Unknown*	28	8.9
Surgery		
Yes	188	60.1
No	125	39.9
Type of surgery		
Modified radical mastectomy	181	96.3
Lumpectomy	3	1
Simple mastectomy	4	1.3
Margin status		
Negative	184	97.9
Positive	4	2.1
Endocrine therapy$		
Yes	123	39.3
No	190	60.7
Anti-HER2 therapy$		
Yes	71	22.7
No	239	76.4
Unknown	3	1
Chemotherapy(first line regimen)$		
CAF/CEF	9	2.9
Taxane based/taxane +anthracyline	241	77.0
Platinum based	17	5.4
Others	20	6.4
Unknown	26	8.3
Bisphosphonate		
Yes	92	29.4
No	221	70.6
OFS*		
Yes	58	18.5
No	255	81.5
Radiotherapy		
Yes	99	31.6
No	184	58.8
Unknown	30	9.6
Viscera metastasis		
Yes	167	53.4
No	146	46.6
Bone metastasis		
Yes	171	54.6
No	142	45.4
CNS metastasis*		
Yes	11	3.5
No	302	96.5
Soft tissue metastasis		
Yes	139	44.4
No	174	55.6
No. of metastasis sites		
≤ 3	288	92.0
> 3	25	8.0
Overall survival(months)		
Median	62	–
95% Confidence interval	45.79–78.21	–

Abbreviation: *OFS = ovarian function suppression; CNS = central nervous system; ^$^Endocrine therapy were subsequent to chemotherapy, anti-HER2 therapy were incorporated into or subsequent to chemotherapy

Table 2 shows a comparison of the clinical and tumor characteristics between the surgery group and the no surgery group. Patients undergoing surgery were significantly more likely to have ≤3 metastasis sites (*p* < 0.001), no visceral metastasis (*p* = 0.002), and lower clinical lymph node stage (*p* = 0.018). A significantly higher proportion of patients in the surgery group have tumor size ≤5 cm (*p* = 0.018), received radiotherapy to the chest wall (*p* < 0.001), canonical endocrine therapy (*p* = 0.001), and ovarian function suppression (*p* = 0.001). There was no significant difference with regard to patients’ and tumors’ characteristics such as age distribution; menstrual status; HER2 status; and HR status; molecular subtypes of tumor; receipt of anti-HER2 therapy; and prevalence of bone, CNS, or soft tissue metastasis (Table 2).

**Table 2:** Comparison of clinical and tumor characteristic between patients who underwent surgery and who did not

Variable	Nonsurgical(*N* = 125)	Surgical(*N* = 188)	*p* value^&^
Age			
≤ 50	83(43.7%)	1107(56.3%)	0.092
> 50	42(34.1%)	781(65.9%)	
Menopause status			
Menses	72(36.8%)	1122(63.2%)	0.144
Menopause	51(45.2%)	663(54.8%)	
Tumor size			
≤ 5 cm	63(36.2%)	1111(63.8%)	***0.018***
> 5 cm	60(50.0%)	560(50.0%)	
Lymph node involvement			
N0	5(17.9%)	223(82.1%)	***0.018#***
N1	21(47.7%)	23(52.3%)	
N2	31(43.7%)	340(56.3%)	
N3	65(50.4%)	664(49.6%)	
HER-2 receptor status			
Amplified	49(37.1%)	783(62.9%)	0.657^£^
No-amplified	67(41.6%)	994(58.4%)	
Not known or equivocal(2+)	49(45.0%)	511(55.0)	
Hormone receptor status			
H+	81 (38.2%)	1131 (61.8%)	0.588
H-	39(41.5%)	555(58.5%)	
Molecular subtype			
Luminal A	19(34.5%)	36(65.5%)	0.194
Luminal B	54(38.3%)	887(61.7%)	
HER2	23(41.1%)	333 (58.9%)	
Triple negative	12 (36.4%)	21 (63.6%)	
Unknown	117 (60.7%)	911 (39.3%)	
Viscera metastasis			
Yes	80 (47.9)	887 (52.1)	***0.002***
No	45 (30.8%)	9101 (69.2%)	
Bone metastasis			
Yes	75(43.9%)	996(56.1%)	0.120
No	50(35.2%)	892(64.8%)	
CNS metastasis*			
Yes	6(54.5%)	5(45.5%)	0.314
No	119(39.4%)	1183(60.6%)	
Soft tissue metastasis			
Yes	58(42.3%)	779(57.7%)	0.297
No	67(38.1%)	1109(61.9%)	
No. of metastatic sites			
≤ 3	107(37.2%)	1181(62.8%)	***< 0.001***
> 3	18(72%)	67(28%)	
Endocrine therapy^$^			
Yes	35(28.5%)	888(71.5%)	***0.001***
No	90(47.4%)	9100(52.6%)	
Anti-HER2 therapy^$^			
Yes	24(33.8%)	447(66.2%)	0.055£
No	98(41.0%)	1141(59.0%)	
Unknown	3(100%)	0	
OFS*			
Yes	12(20.7%)	446(79.3%)	***0.001***
No	113(44.3%)	1142(55.7%)	
Radiotherapy			
Yes	18(18.2%)	781(81.8%)	***< 0.001***
No	81(44.0%)	1103(56.0%)	
Unknown	26(86.7%)	4(13.3%)	

*Abbreviation*: * OFS = ovarian function suppression; CNS = central nervous system; ^$^Endocrine therapy were subsequent to chemotherapy; anti-HER2 therapy were incorporated into or subsequent to chemotherapy; ^&^×2 test, except ^£^Fisher’ exact test

*P* values in bold italic are considered statistically significant

### Univariable and multivariable analysis

Univariable analysis using Cox hazard model showed that patients in the surgery group had lower mortality risk than patients in the no surgery group (HR = 0.53; 95% CI, 0.36–0.78; *p* = 0.001; Table 3). Also, we observed factors: visceral metastasis (yes vs. no *p* = 0.005), age at diagnosis (> 50 vs. ≤50; *p* = 0.026), HR status (HR+ vs. HR−; *p* < 0.001), endocrine therapy (yes vs. no; *p* < 0.001), and radiotherapy (yes vs. no; unknown vs. no; *p* = 0.029) were significant prognostic factors for OS. Multivariable analysis showed that surgery (yes vs. no; *p* = 0.001), HR status (HR+ vs. HR−; *p* = 0.013), and endocrine therapy (yes vs. no; *p* = 0.009) were independent prognostic factors in de novo stage IV breast cancer.

**Table 3:** Cox regression predicting mortality risk for Women With Stage IV Breast Cancer (univariable and multivariable)

Characteristic	Univariable HR^*^(95%CI)	P	Multivariable HR^*^(95%CI)	P
Surgery				
Yes vs No	0.56(0.382-0.820)	***0.003***	0.53(0.36-0.78)	***0.001***
Viscera metastasis				
Yes vs no	1.73 (1.18-2.54)	***0.005***		
Bone metastasis				
Yes vs no	0.91 (0.62-1.32)	0.610		
CNS metastasis				
Yes vs no	1.40 (0.44-4.43)	0.566		
Soft tissue metastasis				
Yes vs no	0.99 (0.68-1.46)	0.977		
Age				
≤50 vs >50	0.65 (0.44-0.95)	***0.026***		
Menopause status				
Menopause vs menses	1.21 (0.82-1.77)	0.344		
Biopsy				
mass resection	1§	0.753		
core needle	1.32 (0.64-2.73)	0.457		
fine needle	1.43 (0.18-11.59)	0.736		
Histology				
ductal	1§	0.824		
lobular	0.66 (0.16-2.69)	0.563		
others	1.11 (0.41-3.05)	0.835		
Tumor size				
>5 cm vs ≤5 cm	1.00 (0.67-1.49)	0.981		
Lymph node involvement				
N_0_	1§	0.259		
N_1_	2.48 (1.00-6.11)	0.049		
N_2_	1.95 (0.80-4.74)	0.142		
N_3_	2.14 (0.91-5.05)	0.081		
HER2 receptor status				
Amplified	1§	0.557		
No-amplified	0.81 (0.55-1.21)	0.304		
Not known or equivocal(2+)	0.77 (0.31-1.93)	0.574		
Hormone receptor status				
+ vs -	0.49 (0.321-0.7233)	***<0.001***	0.57 (0.36-0.89)	***0.013***
No. of metastasis sites				
>3 vs ≤3	1.751 (0.881-3.477)	0.11		
Endocrine therapy^$^				
Yes vs no	0.45 (0.30-0.68)	***<0.001***	0.55 (0.35-0.86)	***0.009***
Anti-HER2 therapy^$^				
No	1§	0.11		
Yes	0.54 (0.325-1.008)	0.035		
Unknown	0.00 (0.00-5.357E+159)	0.959		
Radiotherapy				
No	1§	***0.029***		
Yes	0.61 (0.40-0.94)	0.025		
Unknown	1.35 (0.71-2.57)	0.357		

*Abbreviation*: § Reference; * OFS = ovarian function suppression; CNS = central nervous system; HR = Hazard ratio; ^$^Endocrine therapy were subsequent to chemotherapy; anti-HER2 therapy were incorporated into or subsequent to chemotherapy

*P* values in bold italic are considered statistically significant

### Overall survival

**Fig. 1:**
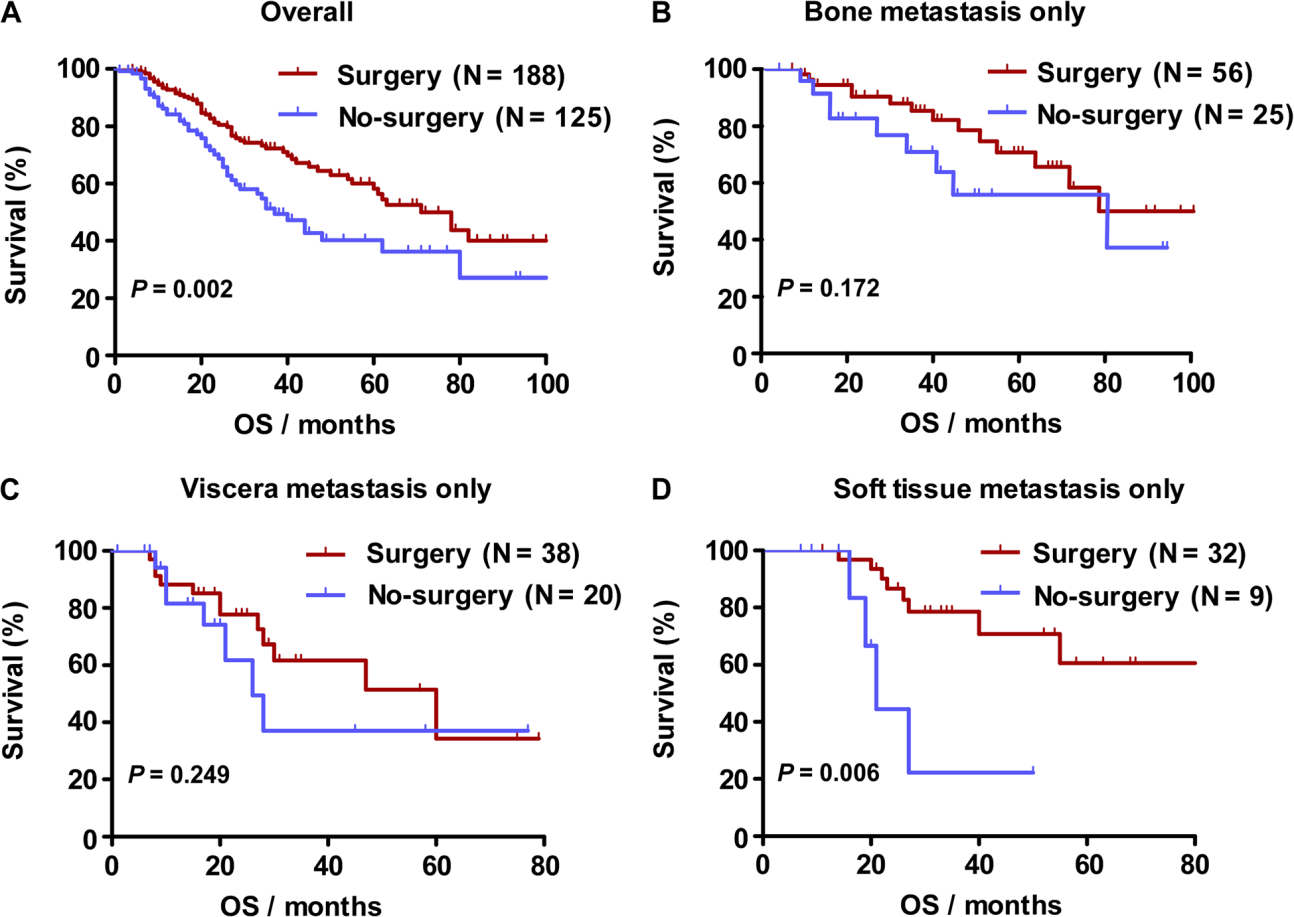
Kaplan-Meier curves estimate overall survival in: a. de novo stage IV breast cancer (Median survival was 78 months in the surgery group vs. 37 months in the no surgery group; log-rank test: *p* = 0.002); b. bone metastasis only (Median survival was 106 months in the surgery group vs. 80 months in the no surgery group; log-rank test: *p* = 0.172); c. viscera metastasis only (Median survival was 60 months in the surgery group vs. 26 months in the no surgery group; log-rank test: *p* = 0.249); d. soft tissue metastasis only (Median survival was 136 months in the surgery group vs. 21 months in the no surgery group; log-rank test: *p* = 0.006)

Figure 1 shows the Kaplan-Meier survival curves. Patients who underwent surgery had significantly better survival than those who did not accept surgery (median survival: 78 months vs. 37 months; *p* = 0.002; Fig. 1a). Surgery did not correlate with better survival in only bone or viscera metastasis patients (median survival: 106 months vs. 80 months; log-rank test: *p* = 0.172; median survival: 60 months vs. 26 months; log-rank test: *p* = 0.249; Fig. 1b-c). In patients with only soft tissue metastasis, local resection of primary tumor could significantly prolong overall survival (median survival: 136 months vs. 21 months; log-rank test: *p* = 0.006).

### Stratified survival analysis

**Fig. 2:**
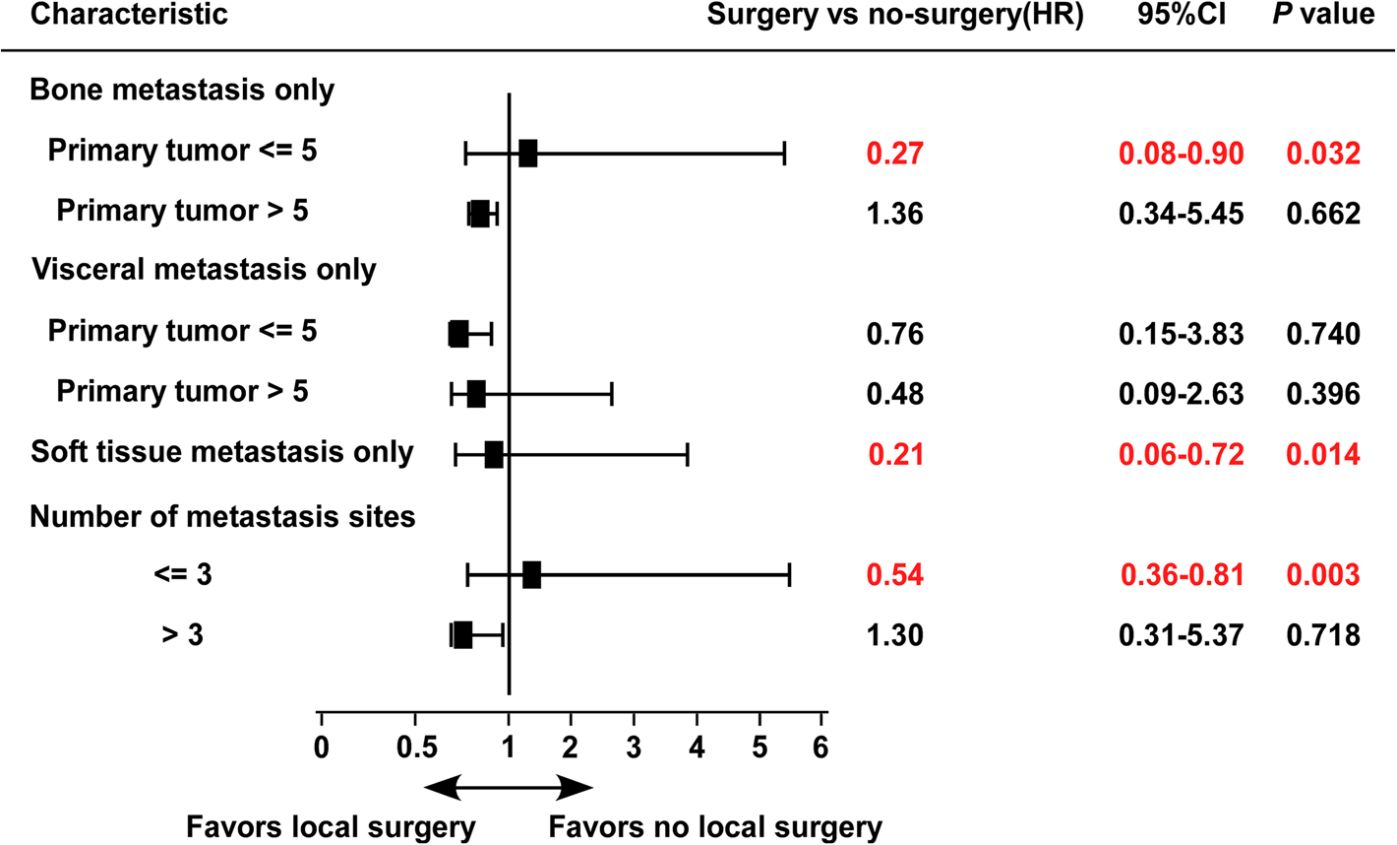
Forest plot of overall survival stratified analysis (the values displayed are unadjusted hazard ratios)

We used stratified survival analysis to characterize the patient who might be expected to benefit from surgery (Fig. 2). Local surgery significantly reduced mortality risk when the primary tumor was ≤5 cm in size in patients with bone metastasis alone (HR = 0.27; 95% CI, 0.08–0.90; Fig. 2); however, surgery provided no benefit when the primary tumor was > 5 cm in size (HR = 1.36; 95% CI, 0.34–5.45). Surgery significantly reduced mortality when there was soft tissue metastasis alone (HR = 0.21; 95% CI, 0.06–0.72) or when there were ≤ 3 metastasis sites (HR = 0.54; 95% CI, 0.36–0.81). No survival benefit was observed following surgery in patients who had > 3 metastasis sites (HR = 1.30; 95% CI, 0.31–5.37), or had visceral metastasis only (irrespective of the tumor size).

## Discussion

This retrospective study was designed to examine how resection of the primary tumor affects survival in patients with de novo stage IV breast cancer and to characterize the population who could be expected to benefit from this surgery. The results showed that resection of the primary tumor could benefit patients with bone metastasis alone (and primary tumor ≤5 cm in size), those with soft tissue metastasis only, and those with ≤3 metastasis sites.

Stage IV breast cancer is considered incurable but treatable [23]. The primary aim of treatment is to delay disease progression and alleviate symptoms. Treatment mostly comprises systemic therapy, including chemotherapy, endocrine therapy, anti-HER2 therapy, and so on; surgery is reserved for tumor bleeding or ulceration [24, 25]. Earlier studies had indicated that the growth of distant metastases could be stimulated by advanced local surgery. Researchers noted that the primary tumor could suppress the growth of distant metastases by secreting angiostatin. Surgical resection reduced angiostatin secretion and also stimulated the release of growth factors, and thus promoted tumor growth [26–28]. However, several retrospective studies have found that local surgery may improve prognosis in breast cancer, renal cell cancer, colorectal cancer, gastric cancer, and melanoma [12, 29–31]. Two experimental study suggested that although surgical resection of the primary tumor may cause transient increase in tumor burden, it substantially reduced overall tumor burden and improved survival by restoring immune responsiveness [32, 33]. It has been suggested that resection of the primary tumor improves survival by both reducing tumor burden and by enhancing sensitivity to chemotherapy [32]. To evaluate the feasibility of local surgery in metastatic breast cancer, several randomized trials have been launched, while two trials have been completed [14, 34]. In 2015, the first trial on the effect of surgery for de novo stage IV breast cancer in India suggested that local surgery did not significantly improve overall survival [4]. Another completed trial (NCT00557986) in Turkey showed that local surgery did not achieve a survival benefit after 3 years of follow-up, but after 5 years follow-up, patients with local surgery achieved a better survival. The effectiveness of local surgery in de novo stage IV breast cancer remains uncertain. Our results, which showed that resection of primary tumor could significantly improve overall survival in de novo stage IV breast cancer, are consistent with the findings of several previous studies [2, 5, 9, 10, 35].

The skeleton is the most common site of metastasis in breast cancer patients [36]. Patients with skeletal metastasis are more sensitive to systemic therapy and have relatively better outcomes than patients with metastasis to other sites [37]. Thus, patients with bone metastasis who are responsive to systematic therapy are likely to gain added benefit from local surgery. Previous studies have found that local surgery lowers mortality in breast cancer patients with bone metastasis [6, 13, 18]. Our study also found that patients with only bone metastasis (and with primary tumor ≤5 cm) benefit from surgery. We also observed a survival benefit following local surgery in patients with only soft tissue metastasis or with ≤3 metastasis sites, and this has not been reported earlier. The underlying mechanism is unclear.

This study had some limitations. This was a single-center retrospective study and, inevitably, a selection bias exists. Our findings need to be validated in prospective multicenter studies on larger cohorts.

## Conclusions

In summary, this study demonstrates a survival benefit following local surgery in de novo stage IV breast cancer patients. Stratified survival analysis showed significant survival benefit in patients with bone metastasis only (with primary tumor ≤5 cm), those with soft tissue metastasis, and those with ≤3 metastasis sites. Thus, in de novo stage IV breast cancer patients who satisfy any of these criteria, it would be reasonable to combine local surgery with systematic therapy.

## Abbreviation

CAF: Cyclophosphamide/doxorubicin/fluorouracil
CEF: Cyclophosphamide/epirubicin/fluorouracil
CNS: Central nervous system
HR: Hormone receptor
OFS: Ovarian function suppression
